# On the use of generative models for demographic inference in malaria vectors from genomic data

**DOI:** 10.1093/g3journal/jkag114

**Published:** 2026-05-14

**Authors:** Amelia Adibe Eneli, Pui Chung Siu, Manolo F Perez, Austin Burt, Matteo Fumagalli, Sara Mathieson

**Affiliations:** School of Biological and Behavioural Sciences, Queen Mary University of London, Mile End Road, London E1 4NS, United Kingdom; School of Biological and Behavioural Sciences, Queen Mary University of London, Mile End Road, London E1 4NS, United Kingdom; Departamento de Biodiversidad y Conservación, Real Jardín Botánico, CSIC, 2 Pl. Murillo, Madrid 28014, Spain; Department of Life Sciences, Imperial College London, Silwood Park, Ascot SL5 7PY, United Kingdom; Department of Life Sciences, Imperial College London, Silwood Park, Ascot SL5 7PY, United Kingdom; School of Biological and Behavioural Sciences, Queen Mary University of London, Mile End Road, London E1 4NS, United Kingdom; The Alan Turing Institute, 96 Euston Road, London NW1 2DB, United Kingdom; Department of Biology, University of Pennsylvania, Philadelphia, PA 19104, United States; Department of Computer Science, Haverford College, Haverford, PA 19041, United States

**Keywords:** malaria parasite, demographic inference, generative adversarial networks, population genetics

## Abstract

Malaria in sub-Saharan Africa is transmitted by mosquitoes from the *Anopheles* genus. Efforts to control the spread of malaria have often focused on these vectors, but little is known about the demographic history of populations and species of *Anopheles* mosquitoes. Here, we adapt and apply an innovative generative deep learning algorithm to infer the joint evolutionary history of *Anopheles gambiae* populations sampled in Guinea and Burkina Faso. We further develop a model selection approach and discover that an evolutionary model with migration fits this pair of populations better than a model without post-split migration. For the migration model, we find that our method accurately captures population genetic differentiation. These findings demonstrate that machine learning and generative models are a valuable direction for future understanding of the evolution of malaria vectors, including the joint inference of demography and natural selection. Understanding changes in population size, migration patterns, and adaptation in hosts, vectors, and pathogens will assist malaria control interventions, with the ultimate goal of predicting nuanced outcomes from insecticide resistance to population collapse.

## Teaser

Efforts to control the spread of malaria have often focused on mosquitoes, but relatively little is known about their evolutionary history. Here, Eneli *et al*. adapt and apply an innovative, generative, deep-learning algorithm to populations sampled from Guinea and Burkina Faso, West Africa. The authors further develop a novel model selection approach and discover that an evolutionary model with migration fits these populations better than a model without migration. Understanding changes in population size, migration patterns, and adaptation in hosts, vectors, and pathogens will assist malaria control interventions, with the ultimate goal of predicting nuanced outcomes from insecticide resistance to population collapse.

## Introduction

### Genetic monitoring of malaria vectors: challenges and opportunities

Malaria is a tropical disease that, in humans, is mainly caused by the *Plasmodium falciparum* parasite transmitted by the female *Anopheles* mosquito. Infected female *Anopheles* mosquitoes introduce parasites through their saliva into the bloodstream of humans they bite (Milner Jr 2018). The World Malaria Report 2024, released by the World Health Organization (WHO), indicates a rise in malaria cases worldwide, with an estimated 263 million cases in 2023, compared to 252 million in 2022 and 247 million in 2021 (Poespoprodjo et al. 2023; Organization 2024). Malaria interventions and elimination efforts have historically relied on vector control, particularly insecticide-based tools such as insecticide-treated nets and indoor residual spraying, which together account for most of the reduction in malaria burden in endemic countries (Killeen et al. 2017). These interventions aim to prevent human–mosquito contact and rapidly kill adult mosquitoes, but their impact is increasingly threatened by widespread resistance to the limited classes of available public health insecticides, especially pyrethroids (Kabula et al. 2024).

Insecticide resistance in mosquitoes is a significant challenge for vector control strategies aimed at reducing mosquito-borne diseases in sub-Saharan Africa. In addition to genetic mechanisms, environmental and operational factors contribute to the development and spread of insecticide resistance in mosquito populations. Environmental factors include local climatic, ecological, agricultural, and urban conditions (Nkya et al. 2013; Owusu et al. 2017), while operational factors refer to the repeated and improper use of insecticides (Hobbs et al. 2023). Genetic resistance to insecticides encompasses target-site, metabolic, cuticular, and behavioral mechanisms. Target-site resistance is changes in proteins targeted by insecticides. A key example is the knockdown mutation (kdr) in the voltage-gated sodium channel gene that confers resistance to pyrethroids and DDT. The kdr mutation occurs in two forms in African Anopheline mosquitoes: kdr-east (L1014S/L995S) and kdr-west (L1014F/L995F) (Grigoraki et al. 2021; Mwagira-Maina et al. 2021; Suh et al. 2023). Metabolic resistance involves changes in genes coding for detoxification enzymes, primarily cytochrome P450s, esterases, and glutathione S-transferases (Adedeji et al. 2020). These enzymes can break down insecticides before they reach their target sites. Cuticular resistance refers to genetic changes that reduce insecticide penetration, often associated with thickening of the cuticle or altered compositions (Wood et al. 2010). Finally, behavioral resistance enables mosquitoes to avoid contact with insecticides, although this mechanism is still poorly understood (Gatton et al. 2013; Carrasco et al. 2019). Consequently, maintaining and further reducing malaria transmission would require broadening the range of vector control approaches. This can be achieved by deploying next generation nets in conjunction with larval source and vector management, while establishing strong systems for the surveillance of insecticide resistance (Böhmert et al. 2024; Tiffin et al. 2025). Effective resistance management requires integrated approaches, including rotating insecticides with different modes of action, using synergists to improve insecticide efficacy, and exploring and employing alternative control methods such as biological interventions, eg fungi and bacteria (Kamareddine 2012). More recently, gene drive vector control strategies that employ genetic engineering are being developed to modify mosquito genomes (Nolan 2021). Field release has not yet been performed, but if successful, it would suppress the number of female mosquitoes to promote population collapse.

The extent to which insecticide-resistance mutations can spread throughout the African continent is still under investigation (Hancock et al. 2022). Therefore, inferring adaptive and neutral demographic histories of mosquito populations using genomic data presents an opportunity to improve our understanding of malaria spread. These inferences range from the estimation of historical population sizes and migration rates to the identification of genetic variants under natural selection (Cheng and Steinrücken 2024). A comprehensive understanding of the genetic history of *Anopheles* mosquitoes would inform robust surveillance and control measures against these vectors, the pathogens they spread, and would help predict their potential future demographic changes. Understanding the interactions between demographic history, population structure, gene flow, and local selection pressures is therefore critical to predict and manage the spread of insecticide resistance in mosquito populations.

The large-scale genomic resources generated by the MalariaGEN consortium have provided valuable information on the demographic history of *Anopheles*. Using ∂a∂i (Gutenkunst et al. 2009), a coalescent-based approach based on the site frequency spectrum, scientists inferred an expansion in populations located north of the Congo Basin that occurred between 7,000 and 25,000 yr ago (Anopheles gambiae 1000 Genomes Consortium 2017). Such expansions could be related to the expansion of human populations as a result of agricultural advances (Li et al. 2014). A more recent signal of population bottlenecks, associated with the widespread use of insecticides, was also observed in some populations (Anopheles gambiae 1000 Genomes Consortium 2017). Other demographic inference methods, such as SMC++ (Terhorst et al. 2017), have been applied to mosquito populations, specifically to the invasive species *Aedes aegypti* (Kent et al. 2025). These results also indicate occurrences of population bottlenecks (of varying severity) and re-expansions in the recent past. In addition, dispersal rates and locations of malaria vectors have been inferred using neural networks (Battey et al. 2020; Smith CC and Kern 2023; Smith CC et al. 2023). Due to the complexity of the *Anopheles* system and the ability of neural network-based methods to uncover subtle signals from noisy data, machine learning methods provide a way forward to understand their historical processes.

### Generative models for population genetic data

Supervised machine learning, and deep learning algorithms specifically, have recently emerged as powerful tools to address some of the most complex questions in population genetics (Schrider and Kern 2018; Korfmann et al. 2023; DeGiorgio et al. 2026; Svetec et al. 2026). Convolutional neural networks, in particular, have been used for inference of natural selection, changes in population size, variation of recombination rate, and time to the most recent common ancestor, among other processes (Flagel et al. 2018; Torada et al. 2019). Deep learning has also been shown to complement traditional simulation-based methods such as Approximate Bayesian Computation (ABC) (Sanchez et al. 2021), while outperforming them for some tasks (Perez et al. 2021). In the context of studies on malaria, machine learning has been deployed to detect genomic signatures of selection in vectors (Xue et al. 2021) and *Plasmodium* parasites (Deelder et al. 2021).

Generative models have recently gained popularity, with the ability to create novel text, images, audio signals, and videos from available data. For a given real data set, a generative model refers to any way of quantifying its *distribution*, which can then be used to create synthetic examples in the style of the data set. For biological applications, generative models are an even more recently introduced technology, but they have already shown promising results. In population genetics specifically, simulated data are used extensively for intuition, validation, comparison of methods, and more recently for training machine learning models. Therefore, developing methods to create more realistic simulated data is very desirable. Early generative models in population genetics have been based on generative adversarial networks (GANs) (Wang et al. 2021; Yelmen et al. 2021; Booker et al. 2023; Smith ML and Hahn 2023; Yelmen et al. 2023). (Yelmen and Jay 2023) provided a comprehensive review of generative models for population genetics.

GANs work by training two models in concert: a generator that creates synthetic examples and a discriminator that predicts whether the examples are real or generated (Goodfellow et al. 2020). Throughout the training process, both models ideally improve so that, in the end, the generator is creating synthetic examples that confuse the discriminator. In some implementations (Yelmen et al. 2021, 2023; Szatkownik et al. 2024), the generator is a neural network, creating artificial genetic data with the same single nucleotide polymorphism (SNP) patterns as real data. This is useful for genomic privacy and downstream association studies or polygenic trait analysis.

In another method, called pg-gan (Wang et al. 2021), the generator is based on an evolutionary model. During training, the goal is to estimate the parameters of this evolutionary model that create data that closely match the real data (from the perspective of the discriminator). Additionally, as the discriminator produces a probability (with closer to 1 meaning more “real” and closer to 0 meaning more “fake”), the trained neural network in pg-gan can be used to identify genomic regions of real data containing features unmodeled in the simulations. For example, regions of real data with a very high discriminator score (ie very unlike neutral simulations) may be under natural selection or display unusual recombination or mutational features. In a recent study (Riley et al. 2024), the pg-gan discriminator is fine-tuned using a transfer learning approach to detect various forms of natural selection. One disadvantage of pg-gan (Wang et al. 2021), is that it produces a point estimate of demographic parameters. To better capture uncertainty, Gower and colleagues developed a variation that uses kernel density estimation to estimate a distribution for each evolutionary parameter, by weighting parameter estimates by their discriminator score (ie how realistic are the resulting simulations) (Gower et al. 2023).

Overall, using a GAN for parameter inference in population genetic applications has several advantages over alternative simulation-based methods. GANs can efficiently explore the parameter space to reach the optimal values, unlike ABC algorithms where simulations must be generated in advance from the entire prior distribution. The CNN discriminator of the GAN can also make use of the complete genotype data, rather than summary statistics only. Summary statistics are frequently slow to compute (often quadratic in the number of sites or the number of haplotypes) and need to be selected or designed for each new application. Traditional MLP or CNN approaches would require simulated training data, making use of the real data only at inference time (unlike GANs which use the real data during training). In some contexts, CNNs have been shown to outperform MLPs, which also have the disadvantage of relying on summary statistics (Isildak et al. 2021). Finally, while there are more modern generative methods available in population genetics (eg diffusion models and transformers), the two-part architecture of GANs allows us to swap out a traditional neural network generator with a custom evolutionary simulator, which would be difficult with any other architecture. In general, there is value in exploring what GAN models can achieve in the space of population genetic inference tasks.

Here, we adapt pg-gan to understand and quantify the demographic history of *Anopheles gambiae* mosquitoes. Specifically, we use pg-gan to detect population splits, effective population size changes, exponential growth, and migration rates. Other authors have used pg-gan for the inference of demography in mosquito populations (Small et al. 2023). As the method was originally developed for and applied to human genetic data, our first objective is to adapt pg-gan to take into account the nuances of mosquito data. We then propose and apply a method to compare competing historical scenarios and estimate demographic parameters using GANs. GANs are used to fit a series of demographic models, then a separate neural network model is designed to discriminate between them. This network can be used to identify the most probable demographic history for real mosquito data. Finally, we define future research directions on the development and use of generative models in population genetics for pathogens and disease-vectors. Our software, called pg-gan-mosquito, is open-source and available at https://github.com/mathiesonlab/pg-gan-mosquito/.

## Materials and methods

### Genetic data and model exploration

The *An. gambiae* complex is composed of at least eight mosquito species, five of which are the primary malaria vectors: *An. gambiae*, *An. coluzzii*, *An. arabiensis*, *An. melus*, and *An. merus* (Charlwood 2019). To assess their genetic diversity, population structure and demographic history, we analyzed genomic data of samples captured in 13 sub-Saharan African countries of two species of the complex *An. gambiae* and *An. coluzzii* and three types of hybrid strains from the Ag1000G Phase 2 open access dataset (Anopheles gambiae 1000 Genomes Consortium 2024), for a total sample size of n=1,142 mosquitoes. Following suggested data filtering (Anopheles gambiae 1000 Genomes Consortium 2017), we assessed population structure using uniform manifold approximation and projection (UMAP) (Diaz-Papkovich et al. 2021).

To show the applicability of pg-gan-mosquito to this system, we focused on a pair of populations, Guinea (GN) and Burkina Faso (BF), due to their genetic similarity and geographic proximity. We retrieved haplotype data from 112 *An. gambiae* samples (31 from GN and 81 from BF) from the MalariaGEN database, Phase 2, following a similar data processing protocol to a previous study (Anopheles gambiae 1000 Genomes Consortium 2017). Specifically, we considered only biallelic variants in chromosomal arms 3L and 3R that passed quality control and were located in heterochromatin states. Furthermore, we filtered out SNPs within the *Gste* gene region, a known target of selection associated with insecticide resistance. The data are phased, and haplotypes are ordered by population. The order of haplotypes within each population does not affect our inference procedure.

We used msprime for all our simulations (Kelleher et al. 2016; Baumdicker et al. 2022). We assumed a mutation rate of $3.5\times 10^{-9}$ per site per generation (Anopheles gambiae 1000 Genomes Consortium 2017) and a recombination rate of $1.45\times 10^{-8}$ per site per generation (Adrion et al. 2020; Lauterbur et al. 2023). We report times in generations, but these can be converted to years using 11 generations per year (Anopheles gambiae 1000 Genomes Consortium 2017). We simulated data under two different previously published demographic models—one with bidirectional migration (*mig*) and one without (*no-mig*) (Anopheles gambiae 1000 Genomes Consortium 2017). See Supplementary Table S1 for the parameter ranges used for each evolutionary parameter of these two models.

We sought to compare our demographic inferences with the previously estimated parameters of these models (Anopheles gambiae 1000 Genomes Consortium 2017) that incorporated only information from the joint site frequency spectrum using the software ∂a∂i (Gutenkunst et al. 2009). Using the ensemble of such previous estimates (Anopheles gambiae 1000 Genomes Consortium 2017) (10 for each model), we computed the median for each parameter of each model, as the model with the lowest akaike information criterion (AIC) score was sometimes an outlier. This procedure formed the *baseline* models with which we compare our inference.

For both real and simulated data, each region consists of 224 haplotypes (GN first, followed by BF) as rows and 72 SNPs as columns. For each SNP, the major allele is encoded as 0 and the minor allele as 1. We do not consider nonsegregating sites, although a site may be nonsegregating when considering each population separately. As a second channel, we feed in inter-SNP distances (duplicated down each column).

### Generative adversarial model

In our GAN model, the generator is an evolutionary model (parameterized by effective population sizes, split times, migration rates, etc.) and the discriminator is a convolutional neural network (CNN). The architecture of the CNN discriminator is modified from the original pg-gan and includes two convolutional layers, a permutation-invariant function, and two fully connected layers. See Supplementary Fig. S1 for a complete specification of the architecture; there are 666,593 discriminator weights learned through training.

In a successful GAN training run, we should observe the following patterns in the loss functions and the accuracy of the discriminator. At the start of training, the discriminator should correctly identify real data from simulations, resulting in high accuracy for both types of data. Here, accuracy is measured as the fraction of regions correctly identified as real or simulated data. The loss of the discriminator should be low (easily able to distinguish real vs. simulated), and the loss for the generator should be high (since its generated data are not “fooling” the discriminator yet). As training progresses, the generator improves the quality of the simulations and reduces its loss, and the discriminator is progressively having difficulties distinguishing real data from simulated data. During the stable competing phase the loss functions have more or less plateaued, although there may still be minor fluctuations in the parameter choices of the generator. Ideally, at the end of the training, the discriminator accuracy is around 50% for both the real and the simulated data, indicating high quality simulations that confuse the discriminator.

To adapt pg-gan for mosquito genetic data, we made several modifications to the input, hyper-parameters, and training procedure. Since mosquitoes have a higher density of genetic variants than humans, we increased the number of SNPs per region from 36 to 72 (to still preserve computational feasibility). Since we have a different sample size in each population, we changed the permutation-invariant collapsing function from sum to mean. We used the AdamW optimizer instead of the regular Adam optimizer for a more robust convergence. Due to the higher number of SNPs per region, we changed the number of units in the first fully connected layer to 160. During pretraining (an initial phase of parameter exploration described below), we set the dropout rate to 0.5 and the learning rate to $1\times 10^{-3}$, and during the main training loop we used a dropout rate of 0.8 and a learning rate of $25\times 10^{-6}$. A larger network combined with a larger dropout rate allows the architecture to be more expressive while avoiding overfitting.

We introduced a pretraining procedure if a successful training run is not observed (see Supplementary Fig. S2 for an example). During pretraining, we explore the hyperparameter space to find an initial configuration that produces simulated data that is easily distinguishable from real data. If the number of pretraining iterations is reached without achieving a sufficient discriminator accuracy, we (a) increase the parameter search space, (b) add filters or hidden units to increase the model capacity, and/or (c) lower the dropout rate while monitoring overfitting. If the discriminator quickly obtains a high accuracy, the dropout rate is increased, or the pretraining is forced to continue.

During training, if the discriminator is overfitting (indicated by very high accuracy) we can reduce the number of training iterations. AdamW also reduces the magnitude of the weights, helping to prevent overfitting (Loshchilov and Hutter 2017). If the discriminator is under-fitting, we increase the model capacity and/or lower dropout rate. Finally, if the discriminator accuracy remains high after pretraining, this could suggest that the evolutionary model is not expressive enough to create simulations matching the real data. In these circumstances, a different or more complex evolutionary model is likely needed.

Using this modified version of pg-gan, called pg-gan-mosquito, we fit two different demographic models. The first model (Anopheles gambiae 1000 Genomes Consortium 2017) (*no-mig*) begins with an ancestral population of size $N_{I}$. At time $T_{G}$ the ancestral population can start to grow or contract (exponentially), with a final size of $N_{F}$ right before splitting. The population split occurs at $T_{S}$, with population sizes $N_{I1}$ and $N_{I2}$ for the two populations, respectively. Both populations can undergo exponential size changes until their final sizes, $N_{F1}$ and $N_{F2}$. The second model (*mig*) has the same structure, but with bidirectional migration after the split (parameter $M_{G}$). At the end of training, we consider the generator’s parameters as our final estimates of these evolutionary parameters. As there is some stochasticity in the GAN training process, for each model, we run training from five different initial parameter sets. We choose the parameters from the best fitting replicate by visual inspection of the loss and accuracy curves over the training iterations.

### Demographic model selection and evaluation

To assess which demographic model is a better fit to the data, we developed a machine learning-based model selection algorithm inspired by previous studies (Fonseca et al. 2021; Kirschner et al. 2022). We trained an additional neural network (with the same CNN architecture as the pg-gan-mosquito discriminator) with data simulated under both models. The task of this network is to perform a binary classification on randomly sampled genomic regions; whether each one is better modeled by the *no-mig* or *mig* demographic history. After training is complete, we feed randomly selected regions of *real* data into the trained network to predict whether their histories are more similar to the *no-mig* or mig model. At the end of this step, the fractions of real random regions assigned to each model provide us with confidence about which model is a better fit to the real data. However, we must be cautious about interpreting these results, as some regions will not display admixture by chance.

For the better fitting model, we also sought to assess whether data simulated under this model resembles real data. To do this, we compared the distributions of commonly used summary statistics of genetic diversity and differentiation (Nielsen 2005), as previously done (Wang et al. 2021). These summary statistics include pairwise heterozygosity (*π*), Watterson’s *θ*, site frequency spectrum, inter-SNP distance, linkage disequilibrium (LD) decay, number of haplotypes and a measure of population genetic differentiation ($F_{ST}$).

As an additional evaluation, we ran pg-gan-mosquito on simulated “real” training data where we know the ground truth parameters. In this way, we can evaluate whether pg-gan-mosquito is able to (a) recover parameters that match the parameters of the training data and (b) create data that match the training data even if (a) is not the case. To this end, we simulated training data under our inferred parameters for the no-mig and *mig* model to create two “real” datasets. For each dataset we trained pg-gan-mosquito 10 times to create replicates. We evaluate the results in terms of both the inferred parameters and the fit of the resulting simulations to the training data. This approach complements the validation of our algorithm based on comparing observed and simulated summary statistics.

## Results

After using UMAP to assess the genetic structure in the *Anopheles* complex, as done in previous studies (Anopheles gambiae 1000 Genomes Consortium 2020; McCann et al. 2024), we confirmed that the samples tend to cluster closely according to their species and country of origin (Supplementary Fig. S3). The mainland populations are tightly clustered in both groups with hybrid forms on the periphery of the *An. coluzzii* cluster. Similarly, *An. gambiae* samples from mainland populations form a large cluster, with the different populations appearing distinctly. Samples from Gabon, Uganda, and the Mayotte island form separate and distant clusters.

**Fig. 1.**
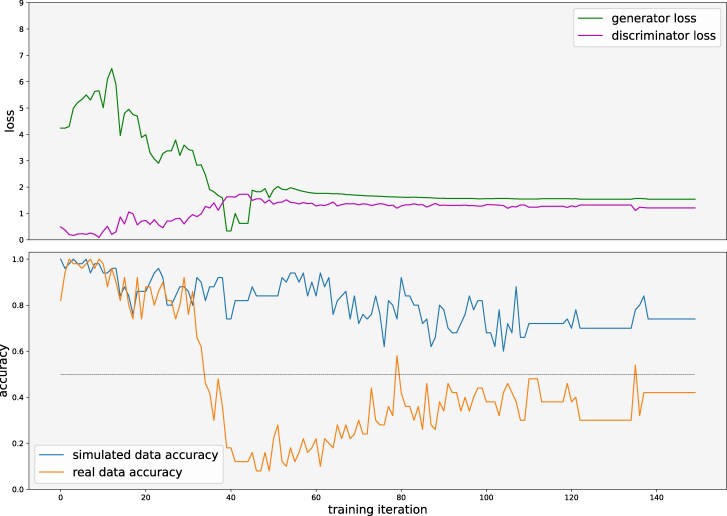
Training run with populations GN and BF under the *mig* model. The top panel shows the generator and discriminator loss functions, which are well-matched by the end of training. The bottom panel shows the discriminator accuracy for simulated data and real data. Accuracy is measured as the proportion of regions the discriminator correctly identifies.

To understand the joint demographic history and divergence of GN and BF *An. gambiae* populations, we fit two different demographic models using pg-gan-mosquito – *no-mig* (no migration after the population split) and *mig* (migration after the population split). Supplementary Fig. S4 and Fig. 1 show successful training runs for the *no-mig* and *mig* models, respectively. Each training iteration represents a mini-batch of data that includes both simulated regions (under the generator’s current parameters) and real regions (chosen randomly throughout the genome). In both cases, we observe that the generator and discriminator losses are well-matched by the end of training. For the accuracies, initially the discriminator is easily able to distinguish real from simulated data, but during training it exhibits some difficulty. At the end of training, the *no-mig* model discriminator displays high, but not perfect, accuracy, indicating an appropriate level of confusion compatible with a successful training. The *mig* model displays accuracies closer to 50% which indicate that the discriminator is confused and the generator produces highly realistic data. In all cases, we take the generator’s parameters at the end of training as our final inference, but early stopping could be explored as an alternative strategy. Supplementary Fig. S2 shows an example of a failed training run, where learning stops, leading to a plateau in loss and accuracy. This type of failed run is not typical (2/5 cases for the *no-mig* model and 1/5 cases for the *mig* model) and strategies for avoiding such outcomes are described in the Materials and Methods section above.

Table 1 shows the point estimates for the model parameters. For both models, we infer a very recent split time ($T_{S}$) with large recent population sizes ($N_{F1}$ and $N_{F2}$).

**Table 1.** Joint population model parameter inference from pg-gan-mosquito.

Name	Description	Baseline *no-mig*	pg-gan-mosquito *no-mig*	baseline *mig*	pg-gan-mosquito *mig*
$N_{I}$	Initial ancestral size	420,699	579,516	415,254	560,796
$T_{G}$	Time of size change of ancestral population	89,497	75,249	91,234	57,254
$N_{F}$	Size of ancestral population before split	9,449,179	2,784,452	9,164,256	7,772,061
$T_{S}$	Time of split	2,243	3,519	2,996	2,265
$N_{I1}$	Initial size of population GN	18,328,570	117,287,221	22,149,704	16,137,900
$N_{I2}$	Initial size of population BF	39,242,967	62,771,288	11,679,596	20,976,274
$N_{F1}$	Final size of population GN	42,056,997	59,092,640	31,103,040	134,237,900
$N_{F2}$	Final size of population BF	42,050,613	189,066,655	19,166,216	218,082,996
$M_{G}$	Migration rate	n/a	n/a	20	27

Model parameter descriptions and baseline inference results are based on (Anopheles gambiae 1000 Genomes Consortium 2017). Time units are in generations, and $M_{G}=2N_{f}m$, where *m* is the bidirectional fractional migration rate per generation.

**Fig. 2.**
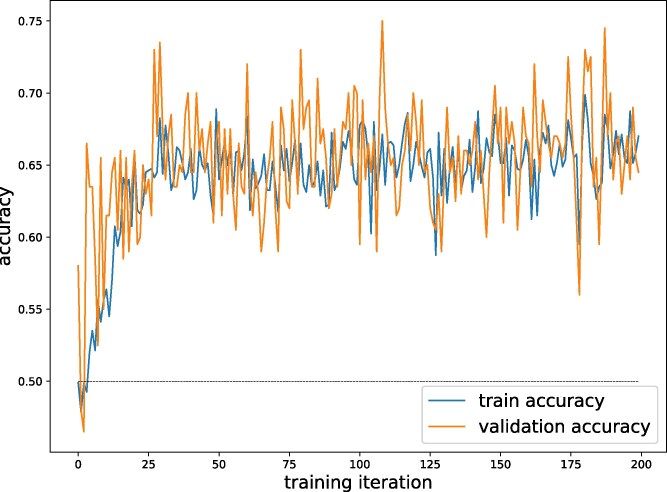
Model selection accuracy curve. We train a discriminator to distinguish data simulated under two models (*no-mig* and *mig*). The accuracy on training and validation data is shown over the training iterations. Although the training is successful, the final accuracy is not very high, indicating these demographic models produce similar data.

To select a demographic model, we used the approach described in the Methods and trained a new CNN using simulated regions from both models. An accuracy curve is shown in Fig. 2, where we plot the training and validation accuracy (both based on simulated data) throughout the training iterations. For each training iteration, each mini-batch includes 50% of regions from the no-mig model and 50% of regions from the *mig* model. Both demographic models produce similar data, as demonstrated by the accuracy starting at around 50% and plateauing between 65% and 70%. As additional testing, we also ran simulated test data through this classification pipeline. For *no-mig* simulations, roughly one-third were classified as *mig*, and for *mig* simulations, roughly one-third were classified as *no-mig*. This result echoes the accuracy curve, suggesting that data from these two models is difficult to distinguish.

After running the GN/BF real data through the model selection discriminator, the fraction of real regions classified as *mig* model is 0.744, while the remaining 0.256 is classified as no-mig. These findings are more extreme than the simulation baseline described above, and thus in line with the observation that these two populations are geographically close. Therefore, we conclude that a model with bidirectional migration explains the observed genetic data better than a model without migration.

**Fig. 3.**
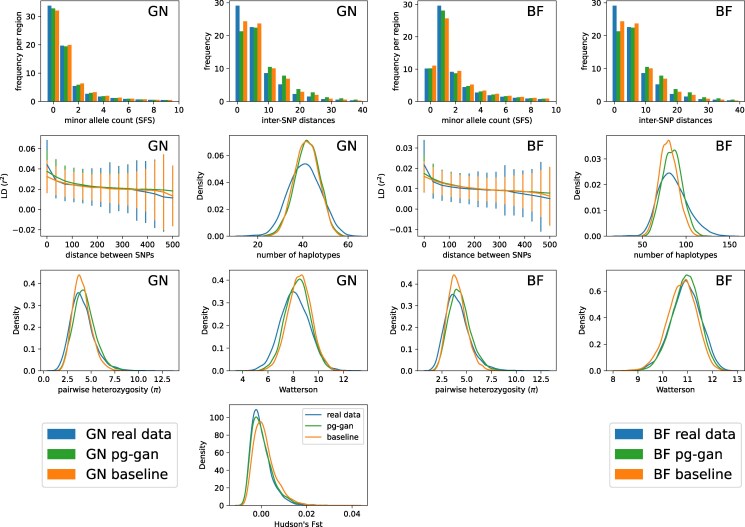
pg-gan-mosquito results for GN and BF populations, fitting a demographic history with migration are shown for three datasets. Blue (“real data”): real data from either the GN or BF population. Green (“pg-gan”): simulations under the parameters inferred by pg-gan-mosquito inference. Orange (“baseline”): simulations under the parameters inferred by ∂a∂i (baseline results from Anopheles gambiae 1000 Genomes Consortium 2017).

To visually assess the match between the data simulated under the inferred parameters of the *mig* model and the real data, we generate distributions of various classical summary statistics. We also plot these distributions under the previously inferred demographic model (*baseline*) (Anopheles gambiae 1000 Genomes Consortium 2017). To obtain each distribution, we simulate many regions under each set of parameters (for the simulated data) and sample random regions from the genome (for the real data). Note that SNPs for each region are a combined set of GN and BF haplotypes. When computing summary statistics for the haplotypes from one of these population, we will observe nonsegregating sites (ie 0 entry in the site frequency spectrum, SFS). These sites indicate the variation was in the other population (or nonsegregating in both with different alleles). Supplementary Fig. S5 and Fig. 3 show these distributions for the *no-mig* and *mig* model, respectively. For the *mig* model, we generally find a similar match between our results (green) and the real data (blue) to the match between the *baseline* model (orange) and the real data (blue).

To quantify how well summary statistics from simulated data match the real data, we compute Wasserstein distances between summary statistic distributions. These values are provided for both *baseline* model and our estimates using pg-gan-mosquito in Supplementary Table S2 for the *no-mig* model, and Supplementary Table S3 for the *mig* model. For the *no-mig* model, we observe that the data simulated under the *baseline* model are generally closer to the real data. On the other hand, for the *mig* model, data simulated under the pg-gan-mosquito model are on average as close to the real data as data simulated under the *baseline* model. We note that $F_{ST}$ is poorly fitted by the *baseline* model, possibly due to the migration rate being capped to its upper limit 20 (Anopheles gambiae 1000 Genomes Consortium 2017). We acknowledge that migration rates are notoriously difficult to estimate (Gower et al. 2023), and we also anticipate some uncertainty in our estimates. Finally, directional migration may provide a better fit than bidirectional gene flow, as modeled here.

**Fig. 4.**
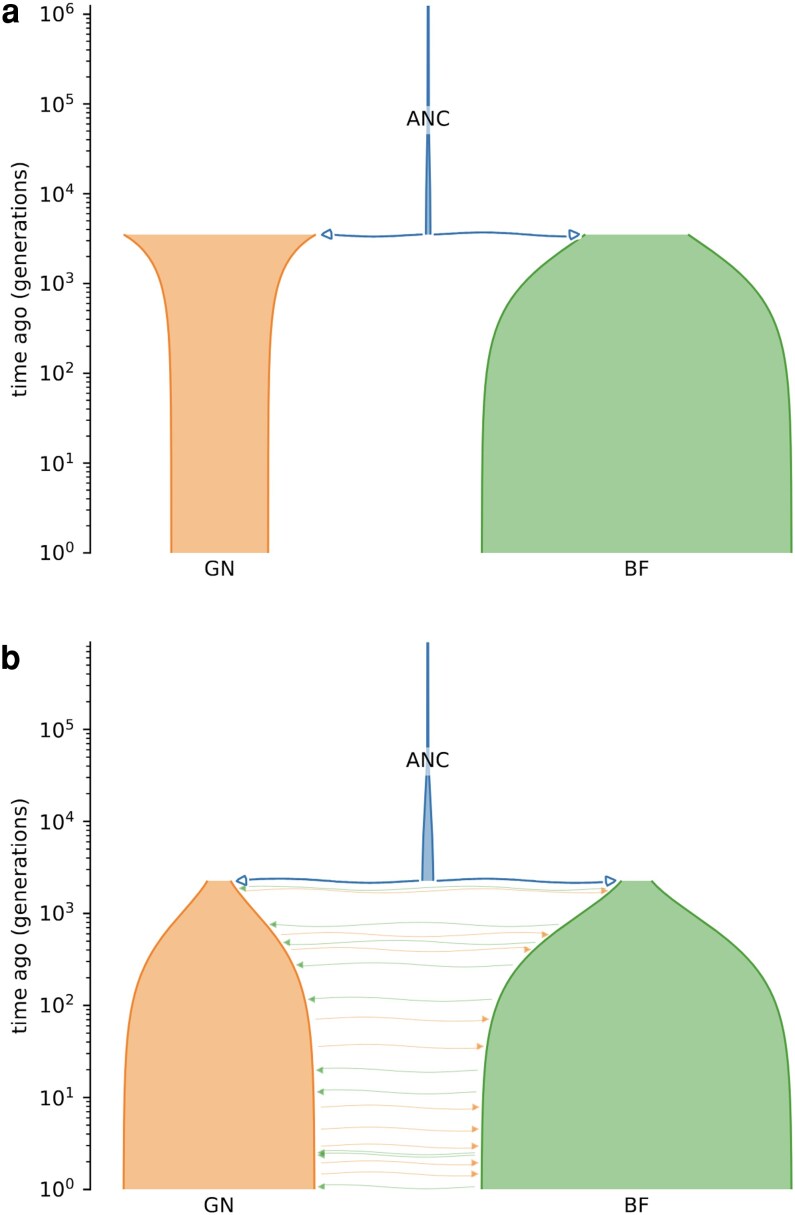
a) pg-gan-mosquito inferred demography for GN and BF using a *no-mig* model. b) pg-gan-mosquito inferred demography for GN and BF using a model with migration (the more likely history based on our model selection results).

Visualizations of both demographic models are shown in Fig. 4. We note that for the migration model, our estimate of $T_{S}$ (time of population split) is much lower than the compared baseline model, and we estimate a higher migration rate (although the baseline model was capped at 20, likely hindering their inference). Given the close proximity of these two populations, we believe that a more recent split time with more migration is compatible with our prior expectations based on the biology and ecology of the species. Additionally, the larger recent effective population sizes could account for the observed diversity between (and within) the two populations. Specifically, these effective population sizes likely result in pg-gan-mosquito SFS and $F_{ST}$ better fitting to the real data than the baseline model.

Finally, we ran pg-gan-mosquito on simulated “real” data where we know the ground truth parameters (described in the Methods section). For each model (*no-mig* and *mig*), we started training from 10 different parameter initializations and selected the parameters from the model with the most desirable loss and accuracy curves by visual inspection. In this experiment, one run failed to train for the *no-mig* data and three for the *mig* data. To assess whether the inference procedure could recover the original parameters, we computed the fractional error as |inferred−true|/true. The results are shown in Supplementary Table S4. In general, we find most parameters are well inferred, aside from recent effective population sizes, likely due to the short region sizes used during inference and/or because of model unidentifiability (ie several parameter sets can create data that are largely indistinguishable). These results demonstrate that our inference of the population split time is more accurate than previous results and that recent effective population sizes may not be exact but within the correct order of magnitude. We also evaluate the fit of the resulting simulated data to the training data. These summary statistic distributions are presented in Supplementary Fig. S6 and show an excellent fit.

## Discussion

In this study, we refine a generative adversarial network algorithm (pg-gan Wang et al. 2021) for use with genetic data from malaria vectors. We apply this demographic inference method to a pair of mosquito populations (GN from Guinea and BF from Burkina Faso) and fit two different evolutionary models, one with post-split migration and one without. We then develop a model selection method based on discriminating between datasets generated under each model, with the goal of identifying which evolutionary history best fits the real data. Our results indicate that a model that includes post-split migration between the GN and BF is most appropriate, consistent with the close geographic proximity of these two populations and previous estimates (Anopheles gambiae 1000 Genomes Consortium 2017). Furthermore, the parameters of our fitted demographic history produce simulated data that are as close to the real data as a previously reported result (Anopheles gambiae 1000 Genomes Consortium 2017).

One limitation of the current implementation of pg-gan-mosquito is that the input must take the form of biallelic SNPs. Although most variants in the *Anopheles* complex are biallelic, we find that on average approximately 23% of sites are triallelic and approximately 3% are tetrallelic. In particular, multiallelism (ie the presence of more than two alleles at a single genetic locus) appears to be highly prevalent in populations that have been sampled near water resources (Supplementary Fig. S7). Swampy vegetation, such as mangroves, may support the lifecycle development (ie eggs, larvae, pupae stages) of the mosquitoes as the water entities of such habitats are less disturbed by anthropogenic activities due to the pneumatophores of the surrounding mangrove vegetation. This, most likely in combination with other pollutants (eg agrochemicals) in the waters, could potentially promote or accelerate the development of insecticide resistant phenotypes (Williams and Hill 2019; Richards et al. 2020). However, it remains unknown whether insecticide resistance is specifically directly correlated with multiallelism (Corbel and N’Guessan 2013; Clarkson et al. 2021). Multiallelism is less pronounced in East and West African populations (relative to Central African populations), where bottlenecks or recent colonization events may have reduced rare variation. In general, future demographic analyses should include information on multiallelic genetic variants to further elucidate recent historical events that affect diversity levels, and machine learning approaches appear to be particularly suitable for this aim. Although multiallelism can be easily included in simulations, it is less obvious how to encode multiallelic sites for the training procedure, with either “one-hot” vectors or different characters of an alphabet (as in language models) being potentially appropriate solutions.

There are many directions for future work in this area, including scaling machine learning-based demographic inference methods to more pairs of populations or groups of populations. Currently, data filtering is performed to retain neutral variation only, but natural selection is still likely to impact our results. Future work could incorporate a distribution of fitness effects, as previous studies aimed to infer demographic histories in the presence of selection (Johri et al. 2021, 2023; Marsh and Johri 2024). Sites under natural selection could also be identified using post hoc analyses of outlier regions that do not fit the inferred demographic history (Riley et al. 2024). A recently proposed and interesting direction is to incorporate spatial features to infer selection targets in malaria vectors (Rehmann et al. 2025). Domain adaptation and transfer learning could also be used to mitigate source/target data mismatch in cases where models have been trained on different species/populations or datasets (Arnab et al. 2025; Cobb and Smith 2025). These techniques could also reduce energy consumption by reducing simulation and training time.

Here, we used UMAP to understand the population structure of malaria vectors, but complementary approaches such as contrastive learning (Thor and Nettelblad 2026), hierarchical soft clustering (Burger et al. 2024), or other supervised nonlinear approaches (Qin et al. 2022) could augment our analyses. Approaches that compare different machine learning methods could help refine the set of evolutionary histories that best explain the data, leading to more robust conclusions about ongoing and future contact between populations.

We envisage that our new implementation, pg-gan-mosquito, will be applicable to a wider range of species with high genetic diversity. By successfully modifying the original implementation, we demonstrate how, in general, generative models are a valuable approach for the demographic inferences of nonmodel species from genomic data. Recent studies have shown that simulation-based approaches can be a suitable framework for inference of evolutionary histories of malaria parasites (Lefebvre et al. 2025), and we argue that our study lays the foundation for future research directions on the joint inference of host, vector and pathogen coevolution using deep learning and synthetic data. Looking ahead to intervention strategies, realistic models of mosquito dispersal and migration between populations could help anticipate the trajectory and tempo of insecticide resistance. A fitted evolutionary history (such as that produced by pg-gan-mosquito) provides a null model for selection scans, which could illuminate which genes contribute to insecticide resistance. Finally, knowledge of the relative effective population sizes in different geographic regions could help us predict the effectiveness of gene drive release strategies and the pace of population collapse.

## Supplementary Material

jkag114_Supplementary_Data

## Data Availability

Mosquito data for the project can be accessed through the MalariaGEN project https://www.malariagen.net/, Phase 2 data. Our software, pg-gan-mosquito is open-source and available on GitHub: https://github.com/mathiesonlab/pg-gan-mosquito/. We also provide instructions on how to use pg-gan-mosquito, which helps researchers of other species who are interested in generative models, demographic inference, and selection of evolutionary models. Supplemental material available at G3 online.
