# Supplementary material for: On the use of generative models for demographic inference in malaria vectors from genomic data
Source: G3 (Bethesda). 2026 May 14;16(7):jkag114. doi: 10.1093/g3journal/jkag114 (PMC13334190; doi:10.1093/g3journal/jkag114)
Supplement: jkag114_Supplementary_Data [file jkag114_supplementary_data.pdf]

Supplementary Material

On the use of generative models for demographic inference in malaria vectors from genomic data

Amelia Adibe Eneli[†,1], Pui Chung Siu[†,1] Manolo F. Perez[2,3], Austin Burt[3], Matteo Fumagalli[1,4], Sara Mathieson[*,5,6]

[1] School of Biological and Behavioural Sciences, Queen Mary University of London, Mile End Road, E1 4NS, London, United Kingdom

[2] Real Jardín Botánico, CSIC, 2 Pl. Murillo, 28014, Madrid, Spain

[3] Department of Life Sciences, Imperial College London, Silwood Park, SL5 7PY, Ascot, United Kingdom

[4] The Alan Turing Institute, 96 Euston Road, NW1 2DB, London, United Kingdom

[5] Department of Biology, University of Pennsylvania, Philadelphia, Pennsylvania, 19104, USA

[6] Department of Computer Science, Haverford College, Haverford, Pennsylvania, 19041, USA

[†] These authors contributed equally.

[*] Corresponding author `smathi@sas.upenn.edu`

Supplementary Tables

name	description	min	max
N_I	initial ancestral size	41,643	3,331,448
T_G	time of size change of ancestral population	50,000	140,000
N_F	size of ancestral population before split	908,628	72,690,248
T_S	time of split	1,000	40,000
N_{I1}	initial size of population GN	602,235	48,178,776
N_{I2}	initial size of population BF	348,034	27,842,680
N_{F1}	final size of population GN	1,951,901	156,152,072
N_{F2}	final size of population BF	4,163,929	333,114,336
M_G	migration rate	0	100

Table S1: Parameter ranges used for `msprime` simulation. The minimum value is 0.1 times the estimates from $\partial a \partial i$ and the maximum is 8 times this value. Time units are in generations. $M_G = 2N_f m$ where m is the bidirectional fractional migration rate per generation (migration is not used for the *no-mig* model). The generator parameters are initially chosen randomly from these ranges, and refined through GAN training.

summary statistic	`pg-gan-mosquito`	baseline
GN minor allele count (SFS)	0.6529	**0.3615**
GN inter-SNP distances	7.5684	**5.0228**
GN distance between SNPs	0.0063	**0.0017**
GN number of haplotypes	3.683	**1.4876**
GN pairwise heterozygosity (π)	1.2472	**0.2023**
GN Watterson	0.4592	**0.3015**
BF minor allele count (SFS)	0.6318	**0.5672**
BF inter-SNP distances	7.5684	**5.0228**
BF distance between SNPs	0.0028	**0.0009**
BF number of haplotypes	7.5718	**5.0602**
BF pairwise heterozygosity (π)	1.2429	**0.2082**
BF Watterson	0.0501	**0.041**
GN/BF Hudson's F_{ST}	**0.0004**	0.0007

Table S2: Wasserstein distances for the *no-mig* model. The left-hand column shows common population genetic summary statistics, computed for each mosquito population separately (GN and BF) and for F_{ST} together. The middle column is the Wasserstein distance between the summary statistic distributions for the real data and data simulated under the `pg-gan-mosquito`-inferred demography. The last column is the Wasserstein distance between the summary statistic distributions for the real data and data simulated under the *∂a∂i baseline* model. The smaller distance in each row is bolded, indicating the closer match to the real data.

summary statistic	`pg-gan-mosquito`	baseline
GN minor allele count (SFS)	**0.2343**	0.4134
GN inter-SNP distances	4.96	**4.1014**
GN distance between SNPs	0.0026	**0.0019**
GN number of haplotypes	1.6676	**1.611**
GN pairwise heterozygosity (π)	0.2646	**0.2046**
GN Watterson	**0.2451**	0.3864
BF minor allele count (SFS)	**0.3449**	0.797
BF inter-SNP distances	4.96	**4.1014**
BF distance between SNPs	0.0012	**0.0011**
BF number of haplotypes	**4.1946**	5.9824
BF pairwise heterozygosity (π)	0.2568	**0.2115**
BF Watterson	**0.0436**	0.1575
GN/BF Hudson's F_{ST}	**0.0005**	0.0016

Table S3: Wasserstein distances for the *mig* model. The left-hand column shows common population genetic summary statistics, computed for each mosquito population separately (GN and BF) and for F_{ST} together. The middle column is the Wasserstein distance between the summary statistic distributions for the real data and data simulated under the `pg-gan-mosquito`-inferred demography. The last column is the Wasserstein distance between the summary statistic distributions for the real data and data simulated under the *∂a∂i baseline* model. The smaller distance in each row is bolded, indicating the closer match to the real data.

error (fractional)	no-mig model	mig model
N_I	0.2215	0.0263
T_G	0.4815	0.1201
N_F	0.2887	0.1004
T_S	0.1742	0.1828
N_{I1}	0.3572	0.1283
N_{I2}	5.673	4.217
N_{F1}	6.399	2.249
N_{F2}	3.480	3.251
M_G	n/a	0.2963

Table S4: Fractional error for each inferred parameter when the "real" data is simulated (and therefore we know the ground truth parameters). Fractional error is computed as |inferred − true|/true. In general we find most parameters are well inferred aside from recent effective population sizes. This is likely due to the short region sizes and/or model unidentifiability.

Supplementary Figures

Layer (type)	Output Shape	Param #
conv2d (Conv2D)	(None, 162, 68, 32)	352
conv2d_1 (Conv2D)	(None, 162, 30, 64)	10,304
max_pooling2d (MaxPooling2D)	(None, 162, 15, 64)	0
flatten (Flatten)	(None, 960)	0
concatenate (Concatenate)	(None, 1920)	0
dropout (Dropout)	(None, 128)	0
dense (Dense)	(None, 320)	614,720
dense_1 (Dense)	(None, 128)	41,088
dense_2 (Dense)	(None, 1)	129

Total params: 666,593 (2.54 MB)

Figure S1: Architecture of the `pg-gan-mosquito` discriminator, a convolutional neural network (CNN). The word `None` here indicates the batch size (number of training or testing regions), which can be flexible. In total there are 666,593 discriminator parameters learned through training.

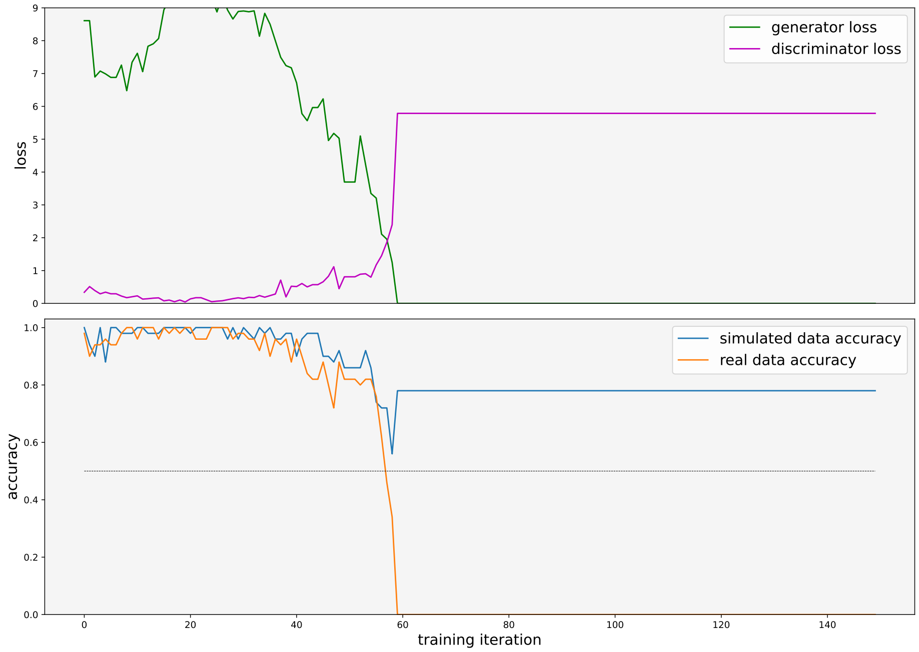

Figure S2: `pg-gan` training run with populations GN and BF under the *mig* model, where training was unsuccessful. The top panel shows the generator (green) and discriminator (pink) loss functions. The bottom panel shows the discriminator accuracy: blue for simulated data and orange for real data. Around training iteration 60, learning stops and the model weights do not change, leading to a plateau in both losses and both accuracies.

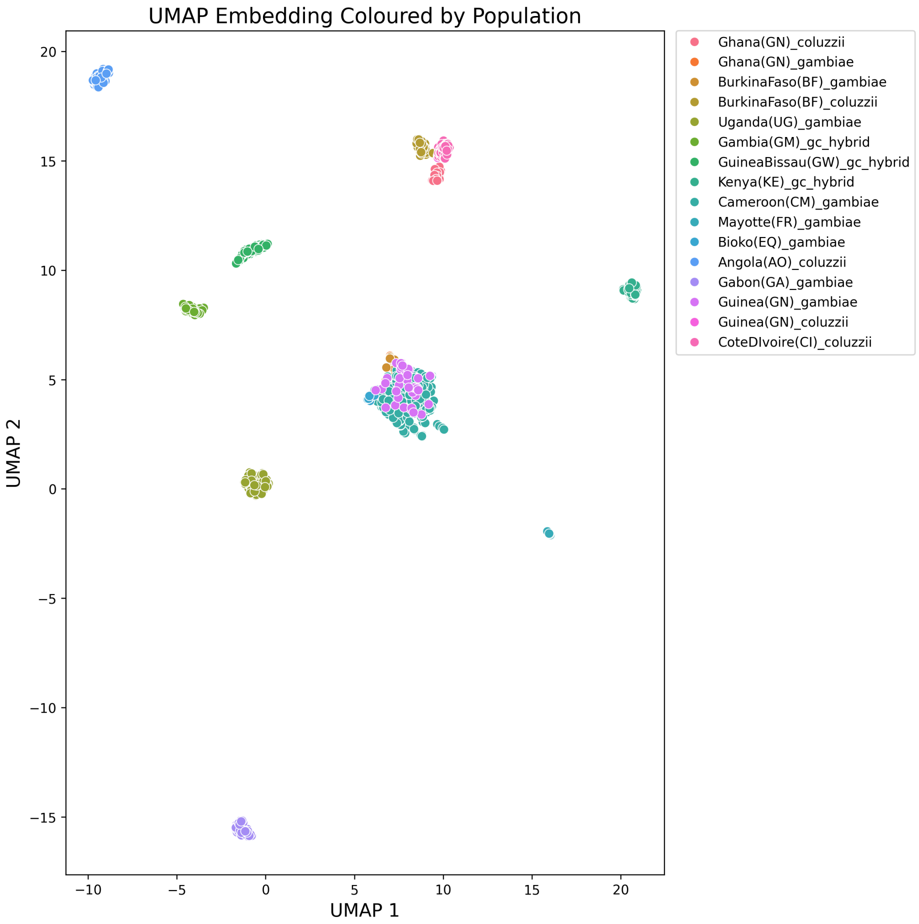

Figure S3: Population structure analysis of mosquitoes using the UMAP technique. Each mosquito represented by a marker. Biallelic SNP data from euchromatic regions of Chromosome arm 3L were projected onto two components denoted as UMAP 1 and UMAP 2.

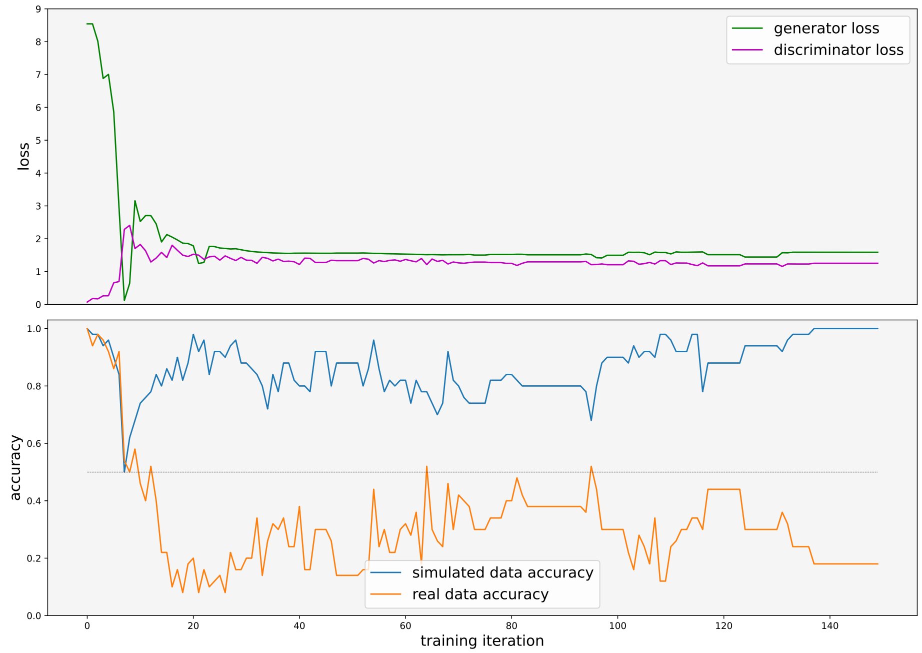

Figure S4: Successful training run with populations GN and BF under the *no-mig* model. The top panel shows the generator (green) and discriminator (pink) loss functions, which are well-matched by the end of training. The bottom panel shows the discriminator accuracy: blue for simulated data and orange for real data. Accuracy is measured as the proportion of regions the discriminator correctly identifies.

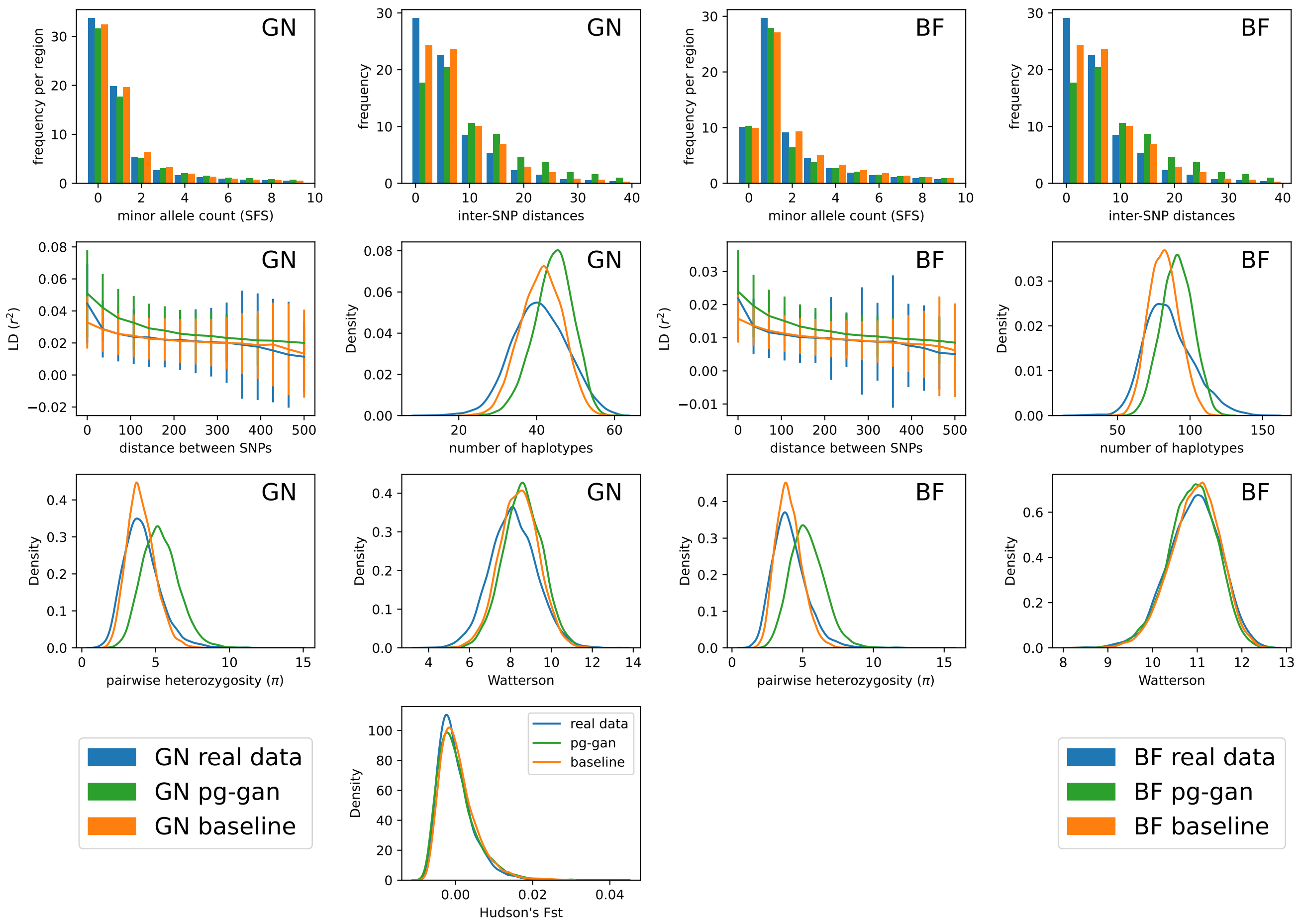

Figure S5: `pg-gan-mosquito` results for GN and BF populations, fitting a demographic history without migration (*no-mig*). Summary statistic distributions are shown for three datasets. Blue: real data from either the GN or BF population. Green: simulations under the parameters inferred by `pg-gan-mosquito` inference. Orange: simulations under the parameters inferred by $\partial a \partial i$ (baseline).

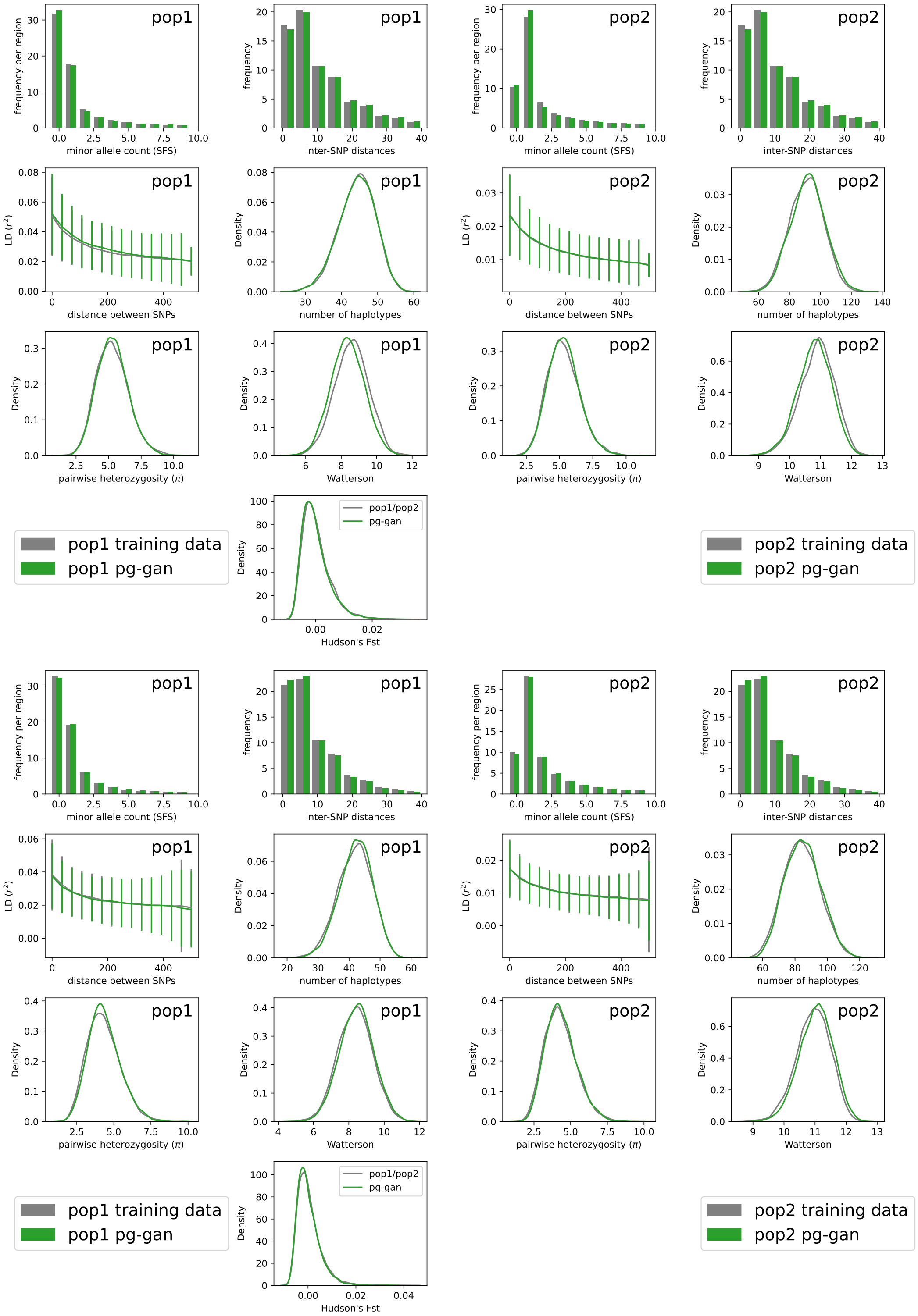

Figure S6: Match of summary statistics between simulated "real" data and `pg-gan-mosquito` parameter inference. Top: fitting data simulated under the *no-mig* model. Bottom: fitting data simulated under the *mig* model.

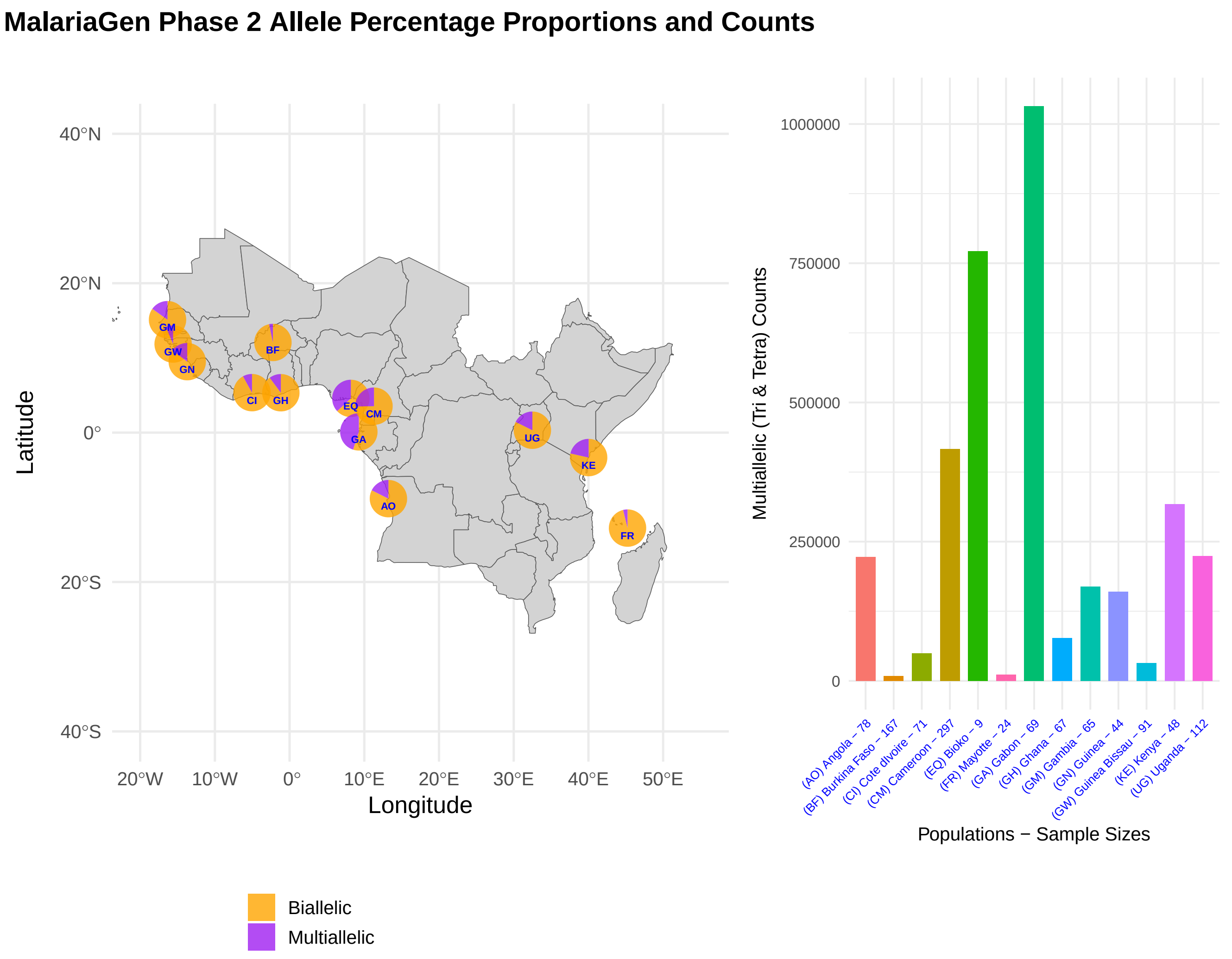

Figure S7: Ag1000G Phase 2 mosquito sample locations. Circle colours denote biallelic (orange) and multiallelic (purple) for triallelic and tetrallelic percentage proportions of each population dataset. The bar chart shows the multiallelic counts for each population.
